# Meta-Analysis of the Detection of Plant Pigment Concentrations Using Hyperspectral Remotely Sensed Data

**DOI:** 10.1371/journal.pone.0137029

**Published:** 2015-09-10

**Authors:** Jingfeng Huang, Chen Wei, Yao Zhang, George Alan Blackburn, Xiuzhen Wang, Chuanwen Wei, Jing Wang

**Affiliations:** 1 Institute of Agricultural Remote Sensing & Information Application, Zijingang Campus, Zhejiang University, Hangzhou, China; 2 Zhejiang Meteorological Service Center, Hangzhou, China; 3 Lancaster Environment Centre, Lancaster University, Lancaster, United Kingdom; 4 Institute of Remote Sensing and Earth Sciences, Hangzhou Normal University, Hangzhou, China; Umeå Plant Science Centre, Umeå University, SWEDEN

## Abstract

Passive optical hyperspectral remote sensing of plant pigments offers potential for understanding plant ecophysiological processes across a range of spatial scales. Following a number of decades of research in this field, this paper undertakes a systematic meta-analysis of 85 articles to determine whether passive optical hyperspectral remote sensing techniques are sufficiently well developed to quantify individual plant pigments, which operational solutions are available for wider plant science and the areas which now require greater focus. The findings indicate that predictive relationships are strong for all pigments at the leaf scale but these decrease and become more variable across pigment types at the canopy and landscape scales. At leaf scale it is clear that specific sets of optimal wavelengths can be recommended for operational methodologies: total chlorophyll and chlorophyll *a* quantification is based on reflectance in the green (550–560nm) and red edge (680–750nm) regions; chlorophyll *b* on the red, (630–660nm), red edge (670–710nm) and the near-infrared (800–810nm); carotenoids on the 500–580nm region; and anthocyanins on the green (550–560nm), red edge (700–710nm) and near-infrared (780–790nm). For total chlorophyll the optimal wavelengths are valid across canopy and landscape scales and there is some evidence that the same applies for chlorophyll *a*.

## Introduction

A pigment is a material that changes the spectral distribution of reflected or transmitted light as the result of wavelength-selective absorption which is determined by the physical properties of the pigment itself. Plant pigments play an important role in light capture, photosystem protection, and in various growth and development functions. The photosynthetic pigments control the amount of solar radiation absorbed by a leaf and thus determine photosynthetic potential and primary production [1,2]. Pigment concentrations are also related to plant stress (excess direct sunlight, UV–B irradiation, low temperature, water stress, nitrogen deficiencies and so on) and senescence (e.g., [3–9]). Therefore, accurate measurements of the temporal dynamics and spatial variations of pigment concentration using remotely sensed data can provide a basis for monitoring physiological and ecological processes [10,11].

The spectral absorbance properties of pigments offer the possibility of using measurements of reflected radiation as a non-destructive method for quantifying pigments. Different approaches have arisen recently to remotely estimate pigment concentrations from a wide variety of wavelengths and sensor types. These studies produced variable results, and none have been demonstrated to have satisfactory performance under all growth and environmental conditions. These inconsistencies may stem from the fact that the experimental results are influenced by a number of factors including different species, experimental conditions and analytical methods used [11].

Recent review articles have attempted to assimilate knowledge in this field of passive optical hyperspectral remote sensing with the sun as energy source. Blackburn [10] reviewed the developing technologies and analytical methods for quantitative estimation of pigment across a range of spatial scales using passive optical hyperspectral remote sensing. Ustin *et al*. [11] appraised the most widely used methodologies for retrieving pigment information with hyperspectral data at the leaf scale. However, it has been demonstrated that traditional qualitative reviewers may subjectively select their preferred studies when faced with conflicting results on a single question [12]. In contrast, it has been argued that meta-analysis can take the results from primary research articles and quantitatively analyze and synthesize these data in an attempt to arrive at more robust conclusions. As such, meta-analysis review papers make the shift from a narrative-driven to a data-driven approach [13,14].

Glass [15] published the first article to lay out the essential rationale of meta-analysis. As a fully general set of methods, meta-analysis has been widely applied to the integration of literatures in many areas of empirical science, including ecology [14]. This form of analysis has, for example, been used to determine the response of biodiversity to intensive biomass production, the effects of elevated CO_2_ on plant–arthropod interactions, the influence of plant invasion on carbon and nitrogen cycles and the causes and consequences of variations in leaf mass per area [16–19]. Today, many findings and advances are being made not only by those who do primary research studies, but also by those who use meta-analysis to discover the latent meaning of existing research literatures [13]. Recently, meta-analysis has been employed in remote sensing research. Garbulsky *et al*. [20] performed a meta-analysis to assess the use of the photochemical reflectance index (PRI) as an indicator of radiation use efficiencies at the leaf, canopy and ecosystem scales for different time scales and vegetation types. Zolkos *et al*. [21] conducted a meta-analysis of publications on LiDAR remote sensing estimation of terrestrial aboveground biomass. These investigations show that meta-analysis can be used to systematically integrate the results from a collection of studies, and through statistical comparison, assess the relationships between remotely sensed measurements and variables of interest.

Here, a meta-analysis of data from a wide selection of studies reporting the passive optical hyperspectral remote sensing of pigments was used to quantify the development of this scientific field, identify optimal wavelengths for retrieval of individual pigments and evaluate the strength of the relationships between pigment concentration and remotely sensed data across pigment types and scales.

## Materials and Methods

### 2.1 Study selection and data extraction

Databases of Elsevier, Springer and Web of Science, licensed to Zhejiang University, were used for source data from inception to August 2014. The following key words were used: pigment, chlorophyll, carotenoids, carotene, xanthophyll, anthocyanins, anthoxanthin in combination with the terms reflectance, estimation, quantification, retrieval, prediction and remote sensing. More than 4500 citations were collected as a result of this initial search.

Then the abstracts of these articles were reviewed and considered for inclusion in the meta-analysis. The following criteria were applied to ensure homogeneity in methodology. First, the studies had to include a chemical measurement of pigment concentration (total chlorophyll, chlorophyll *a*, chlorophyll *b*, carotenoids, xanthophyll, carotene or anthocyanins). Second, the article had to report the quantification of pigments using remotely sensed data. Third, the authors must have provided the following statistical information: (1) coefficient of determination for the relationships between pigment concentration and remotely sensed measurements; (2) the wavelength(s) used to estimate pigment concentration; and (3) training sample sizes.

**Fig 1:**
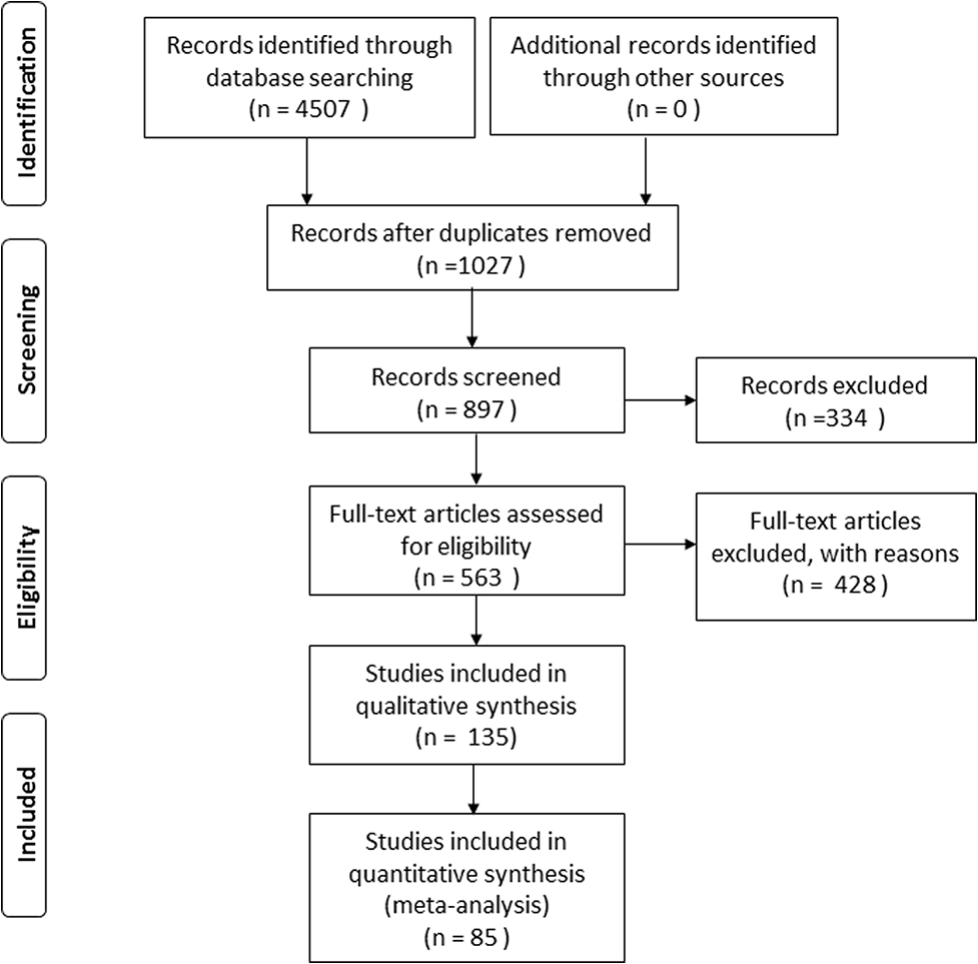
Selection of studies for inclusion in the meta-analysis.

Based on the first two decision rules, 135 articles were selected. According to the final criterion, 50 studies were excluded because of insufficient statistical information. Finally, 85 articles were used in the meta-analysis, which reported results at different spatial and temporal scales and from a wide range of vegetation types between 1977 and 2014. The number of studies selected at various stages is shown in the flow diagram in Fig 1. Some studies reported multiple results for different pigment types or vegetation types. Different types of sensors were used in these studies, from spectrophotometers and hand-held spectroradiometers to satellite sensors. All the sensors were working in reflectance mode. Within the selected articles 44 were working at the leaf scale, 21 at the canopy scale, 15 at the landscape scale, 2 at the leaf and canopy scales, 1 at the leaf and landscape scales, and 2 covered the leaf, canopy and landscape scales. The term “canopy” refers to either a single plant or a monospecific stand where the experimental results are influenced by a number of controlling factors, such as orientation of leaves (leaf angle distribution; *LAD*), variations in number of leaf layers (*LAI*), presence of non-leaf elements, multiple scattering and areas of shadow [10,22], the term “landscape” refers to a mixed-species stand where the reflectance spectrum from airborne and spaceborne sensors is subject to even more controlling factors, such as atmospheric conditions, instrucment sensitivity (signal-to-noise ratio) and spatial resolution. In total, the sample size from all the selected studies is 16100. The Preferred Reporting Items for Meta-Analyses is shown in S1 PRISMA Checklist.

Relevant information was extracted from each study in the final set: ① scales (leaf, canopy, landscape), ② pigment types, ③ species, ④ wavelengths, ⑤ coefficient of determination, ⑥ sample sizes, ⑦ sensors, ⑧ authors and ⑨ year of publication. In order to reduce human error in data extraction and coding, two sets of reviewers independently screened articles in accordance with those inclusion criteria discussed above, evaluated the quality and extracted the data from the eligible studies. The results from one group were cross-checked by the other group. Divergences of opinion about article selection and data extraction were settled by discussion. Table 1 is a summary of the studies contained in this research. This list is not exhaustive but it does cover most papers published related to quantification of pigments using remotely sensed data that met the selection criteria. Table 2 provides a statistical summary of the data extracted from the studies included in the meta-analysis.

**Table 1:** A summary of the studies contained in this research that linked remotely sensed data with pigment. Specrad = spectroradiometer; Specpho = spectrophotometer; Chl tot = total chlorophyll; Chl a = chlorophyll *a*; Chl b = chlorophyll *b*; Cars = carotenoids; Anths = anthocyanins.

Scale	Pigment Type	Year	Species	Sensor	Reference
leaves	Chl tot	1992	Amaranthus tricolor	Specpho	[23]
leaves	Chl tot	1995	Slash pine	Specrad	[24]
leaves	Chl tot	1995	Bigleaf maple	Specrad	[25]
leaves	Chl tot	1996	Horse Chestnut, Norway maple,Cotoneaster, Tobacco	Specpho	[26]
leaves	Chl tot	1996	Norway Maple, Horse Chestnut	Specpho	[27]
leaves	Chl tot	1997	Norway Maple, Horse Chestnut, Fig, Cotoneaster, Tobacco,Oleander, Hibiscus, Vine, Rose	Specpho	[28]
leaves	Chl tot	1998	Tobacco, Horse Chestnut, Cotoneaster	Specpho	[29]
leaves	Chl tot	1999	Beech tree, Elm tree,Wild vine shurb	Specpho	[30]
leaves	Chl tot	1999	Bragg Soybean	Specrad	[31]
leaves	Chl tot	2002	53 species	Specrad	[32]
leaves	Chl tot	2002	Paper birch	Specrad	[33]
leaves	Chl tot	2003	Bigleaf Maple, Horse Chestnut, Wild vine, Beech	Specpho	[34]
leaves	Chl tot	2005	Cotton	Specrad	[35]
leaves	Chl tot	2007	Winter wheat	Specpho	[36]
leaves	Chl tot	2012	15 different species(Beech, Fraxinus lanuginosa, Acer Japonicum, Magnolia obovata and so on)	Specrad	[37]
leaves	Chl tot	2014	Douglas fir	Specrad	[38]
leaves	Chl a	1994	Norway Maple, Horse Chestnut	Specpho	[39]
leaves	Chl a	1994	Norway Maple, Horse Chestnut	Specpho	[40]
leaves	Chl a	1996	Norway Maple, Horse Chestnut	Specpho	[41]
leaves	Cars/Chl tot	1977	Cantaloupe, Corn, Spinach Cotton, Cucumber, tobacco, Head lettuce, Grain sorghum	Specpho	[42]
leaves	Cars/Chl tot	1992	Sunflower	Specrad	[43]
leaves	Cars/Chl tot	1999	Norway Maple, Potato, Lemon, Apple, Coleus	Specpho	[7]
leaves	Cars/Chl tot	2006	24 species of woody trees and shurbs	Specpho	[44]
leaves	Anths/Cars/Chl tot	1999	Quercus agrifolia, Pseudotsuga menziesii	Specpho	[45]
leaves	Anths/Cars/Chl tot	2003	Apple	Specpho	[46]
leaves	Anths/Cars/Chl tot	2004	Norway maple, Maize, Dogwood,Horse chestnut, Second-flush beech, Wild vine shrub, Cotoneaster, Pelargonium zonale	Specpho	[47]
leaves	Chl tot/Anths	2014	Chilean strawberry	Specrad	[48]
leaves	Cars/Chl a/ Chl b	1992	Soybean	Specrad	[49]
leaves	Cars/Chl a/Chl b	1998	Beech, Oak, Maple, Sweet chestnut	Specrad	[50]
leaves	Cars/Chl a/Chl b	2005	Rice	Specrad	[51]
leaves	Chl tot/Chl a/Chl b	1999	Norway Maple, Horse Chestnut, Beech, Oak	Specrad	[52]
leaves	Chl tot/Chl a/Chl b	2001	Croton, Elaeagnus, Japanese pittosporum,Benjamin fig	Specrad	[53]
leaves	Chl tot/Chl a/Chl b	2010	Flowering cherry	Specrad	[54]
leaves	Chl tot/Chl a	1996	Tobacco	Specpho	[55]
leaves	Chl tot/Chl a	1999	Eucalyptus	Specrad	[56]
leaves	Cars	2002	Norway maple, Horse chestnut,Second-flush beech	Specpho	[57]
leaves	Cars	2009	Scot pine	Specpho	[58]
leaves	Cars	2011	Bur oak, Sugar maple, LOPEX database	Specrad	[59]
Scale	Pigment Type	Year	Species	Sensor	Reference
leaves	Anths	2001	Norway maple, Cotoneaster, Dogwood	Specpho	[60]
leaves	Anths	2009	Grapevine	Specrad	[61]
leaves	Anths	2009	European hazel, Siberian dogwood, Norway maple, Virginia creeper	Specpho	[62]
leaves	Anths	2011	Grapevine	Specrad	[63]
leaves	Anths	2011	Sweet cherries	Specpho	[64]
leaves	Anths	2011	Norway maple, Horse chestnut, Beech,Virginia creeper, Dogwood	Specpho&specrad	[65]
Leaves/canopy	Chl tot	2009	Maize	Specpho	[66]
Leaves/canopy	Chl tot	2013	Irrigated maize	Specrad	[67]
Leaves/landscape	Chl tot	2014	Black Spruce, Sugar maple	Specrad&MERIS	[68]
Leaves/canopy/landscape	Chl tot	2010	Winter Wheat, Winter Rapeseed	Specrad	[69]
Leaves/canopy/landscape	Cars/Chl tot	2000	Sugar maple	Specrad	[70]
canopy	Chl tot	1990	Slash pine	Airborne spectro	[1]
canopy	Chl tot	1994	pepper	Specrad	[71]
canopy	Chl tot	2005	Maize, Soybean	Specrad	[72]
canopy	Chl tot	2006	Rice	Specrad	[73]
canopy	Chl tot	2007	Cotton	Specrad	[74]
canopy	Chl tot	2008	Winter wheat, Corns	Specrad	[75]
canopy	Chl tot	2008	Heterogeneous grassland	Specrad	[76]
canopy	Chl tot	2008	Heterogeneous grassland	Specrad	[77]
canopy	Chl tot	2008	Corn, Cotton	Specrad	[78]
canopy	Chl tot	2010	Rice	Specrad	[79]
canopy	Chl tot	2011	Rice	Specrad	[80]
canopy	Chl tot	2012	Potato, Grassland	Specrad	[81]
canopy	Chl tot	2013	Irrigated maize	Specrad	[82]
canopy	Chl tot	2014	Winter wheat	Specrad	[83]
canopy	Chl a	2003	Rice	Specrad	[84]
canopy	Chl a	2007	Winter Wheat	Specrad	[85]
canopy	Chl a/Chl b	2004	Winter wheat	Specrad	[86]
canopy	Chl tot/Chl a	2006	Wheat	Specrad	[87]
canopy	Cars/Chl tot	2010	Tall fescue	Specrad	[88]
canopy	Cars	2008	Kermes oak	Specrad	[89]
canopy	Cars	2008	Douglas fir	Specrad	[90]
landscape	Chl tot	2002	Corn	CASI	[91]
landscape	Chl tot	2003	Eucalypt	CASI-2	[92]
landscape	Chl tot	2004	Jack pine	CASI	[93]
landscape	Chl tot	2004	Douglas fir	MERIS	[94]
landscape	Chl tot	2007	Corn, Wheat	CASI	[95]
landscape	Chl tot	2008	Rice, Cotton	EO-1	[96]
landscape	Chl tot	2008	Garlic, Alfalfa, Onion, Sunflower, Corn, Potato, Wheat, Vineyard, Sugar beet	PROBA/CHRIS	[97]
landscape	Chl tot	2010	Flax, Tea, Chestnut, Corn, Potato, Pine, Bamboo	EO-1	[98]
landscape	Chl tot	2010	Garlic, Onion, Corn, Alfalfa, Sugar beet, Sunflower, Potato, Vineyard, Wheat	PROBA/CHRIS	[99]
landscape	Chl tot	2014	London plane, Canary Island date palm, European nettle tree, White mulberry	CASI	[100]
landscape	Chl a	2004	Winter Wheat	AVIS	[101]
landscape	Cars/Chl tot	2002	Quercus petrea, Pinus sylvestris	CASI	[102]
landscape	Chl a/Cars	2005	Rice	PHI	[103]
landscape	Cars/Chl tot/Chl a/Chl b	2008	Aspen, Birch, Spruce, Balsam fir	CASI	[104]
landscape	Anths	2009	Austrocedrus chilensis forest	Hyperion	[105]

**Table 2:** Summary statistics for the selected studies and extracted data for different pigment types at leaf, canopy and landscape scales.

Scale	Pigment type	Number of studies	Number of effect sizes	Total sample size	Number of wavelengths
leaves	Chl tot	34	53	6431	131
	Chl a	11	23	1595	53
	Chl b	6	10	860	24
	Cars	14	15	1381	40
	Anths	10	17	1752	43
canopy	Chl tot	20	23	1146	55
	Chl a	4	4	162	6
	Chl b	1	1	35	0
	Cars	3	2	45	7
	Anths	0	0	0	0
landscape	Chl tot	15	17	1883	46
	Chl a	3	3	153	6
	Chl b	1	1	24	2
	Cars	3	3	573	4
	Anths	1	1	60	2

### 2.2 Statistical analysis of effect size

#### 2.2.1 The calculation of effect size for each study

The coefficient of determination (R^2^) was used to evaluate the strength of relationships between spectral reflectance and pigment concentration in each article we selected. The value of R^2^, however, is affected by the number of selected wavelengths. The more wavelengths included in the model, be they relevant or not, the larger would be the R^2^ [106]. The increase of R^2^ is not without cost. The increasing number of selected wavelengths reduces the degrees of freedom, which reduces model robustness. The adjusted coefficient of determination was applied to correct for the degrees of freedom:

$$R_{A}^{2}=1-(1-R^{2})\frac{n-1}{n-k}$$ (1)

where *n* is the sample size for each study, *k* is the number of independent variables in the linear or nonlinear model. Eq (1) shows that $R_{A}^{2}$ is always smaller than *R* ^2^ when *k* > 1, which means the growth rate of $R_{A}^{2}$ is lower than that of *R* ^2^ as the number of parameters increase. This result is straightforward and it has been shown that when the added parameter explains a significant amount of the behavior of the dependent variable, $R_{A}^{2}$ will increase; otherwise, $R_{A}^{2}$ will decrease [107]. So $R_{A}^{2}$ was chosen as the effect size statistic, the variance of effect size is calculated as [108]:

$$V_{i}=\frac{{\left(1-R_{A}^{2}\right)}^{2}}{n-1},$$ (2)

The resulting data set was categorized by pigment type at the scales of leaf, canopy and landscape to allow comparison.

#### 2.2.2 Test of heterogeneity for effect sizes

It is important to assess the heterogeneity among the results from a collection of studies before computing the mean effect size [109]. Basically, there are two possible sources of heterogeneity in meta-analysis: methodological heterogeneity and statistical heterogeneity. To ensure homogeneity in methodology, we applied a series of criteria to identify the studies to be used in the meta-analysis (as described in section 2.1). Here the *I* ^2^ statistic was used to test for the statistical heterogeneity. The *I* ^2^ statistic measures the extent of true heterogeneity dividing the difference between the result of the *Q* test and its degrees of freedom by the *Q* value itself [110]:

$$I^{2}=100\%\times \frac{Q_{tot}-df}{Q_{tot}}$$ (3)

where *df* = *N*_*tot*_−1, *N*_*tot*_ is the total number of effect sizes from all the selected studies, *Q*_*tot*_ is computed as [111]:

$$Q_{tot}=\sum_{i=1}^{N_{tot}}W_{i}E_{i}^{2}-\frac{{\left(\sum_{i=1}^{N_{tot}}W_{i}E_{i}\right)}^{2}}{\sum_{i=1}^{N_{tot}}W_{i}}$$ (4)

where Wi=1Vi, *E*_*i*_ is adjusted coefficient of determination ($R_{A}^{2}$).

The *I* ^2^ statistic can be interpreted as the percentage of heterogeneous component in the total variability of effect size (*Q*_*tot*_), so the larger the *I* ^2^ statistic is, the stronger the heterogeneity is. If *I* ^2^ exceeds 50%, the null hypothesis of homogeneity is rejected. The *I* ^2^ statistic for different pigments at different scales were calculated, all the results were lower than 50%, the null hypothesis of homogeneity for this study was accepted.

#### 2.2.3 The calculation of mean effect size for different pigments at different scales

In contrast to studies based on original data, the unit of meta-analysis is the individual research study. Distinctive aspects of data analysis follow from this difference. The first complication is that the studies incorporated into the meta-analysis generally use different sample sizes and this controls the statistical properties of effect sizes [112]. From a statistical perspective, larger sample studies have less sampling error than smaller sample studies, thus more weight should be assigned to larger sample studies in the computation of the mean effect size. The other complication is inter-study variability, which is caused by the influence of an indeterminate number of characteristics that vary among the studies.

Considering the two sources of variability discussed above, a random effects model was used to compute the weighted mean of $R_{A}^{2}$ for different pigment types. In contrast to a fixed effects model, the weight applied to each effect size in a random effects model must represent both subject-level sampling error and the additional random variance component [112]. As such, the mean effect size becomes a reasonable estimate of the true strength of the effect in the population. Because of the generality of the random effects model, it is the preferred strategy in meta-analysis [113]. The mean effect size is computed as:

$$M_{rand}=\frac{\sum_{i=1}^{N_{p}}W_{i(rand)}E_{i}}{\sum_{i=1}^{N_{p}}W_{i(rand)}}$$ (5)

The variance is:

$$V_{rand}=\frac{1}{\sum_{i=1}^{N_{p}}W_{i(rand)}}$$ (6)

where $W_{i(rand)}=\frac{1}{V_{i}+\sigma^{2}}$, $\sigma^{2}=\frac{Q_{p}-(N_{p}-1)}{\sum_{i=1}^{N_{p}}W_{i}-\frac{\sum_{i=1}^{N_{p}}W_{i}^{2}}{\sum_{i=1}^{N_{p}}W_{i}}}$, Wi=1Vi, $Q_{p}=\sum_{i=1}^{N_{p}}W_{i}E_{i}^{2}-\frac{{\left(\sum_{i=1}^{N_{p}}W_{i}E_{i}\right)}^{2}}{\sum_{i=1}^{N_{p}}W_{i}}E_{i}$, is adjusted coefficient of determination ($R_{A}^{2}$) and *N*_*p*_ is the total number of effect sizes for a specific type of pigment at each different scales (Table 2). Using this approach the mean effect size of Chl tot, Chl a, Chl b, Cars and Anths at the scales of leaf, canopy and landscape were calculated.

A confidence interval gives the range of values within which the mean effect size is likely to be, it is useful in indicating the degree of precision of the estimate of the mean effect size. A 95% confidence interval is subsequently calculated as follows:

$$Conf_{95}=M_{rand}\pm 1.96SE_{rand}$$ (7)

where $SE_{rand}=\sqrt{V_{rand}}$. If the confidence intervals of multiple mean effect sizes donot overlap, then there are significant differences between these mean effect sizes.

### 2.3 Optimal wavelengths for pigment quantification

A large number of narrow-band indices were proposed to measure plant pigments in the selected articles. These narrow band indices include difference vegetation index (NBDVI), ratio vegetation index (NDRVI), normalized difference vegetation index (NBNDVI), anthocyanin reflectance index (ARI), soil-adjusted vegetation index (SAVI), perpendicular vegetation index (PVI) and so on. The wavelengths used in these studies are different and there is lack of agreement on optimal wavelengths for pigment quantification.

Histograms and quantile plots were used to identify the optimal wavelengths for individual pigment quantification at different scales. The histogram partitions the data distribution of wavelengths into subsets of 10nm width. This enabled us to provide an overview of suitable wavelengths, which is difficult to achieve if the analysis is performed at higher spectral resolutions. Also this approach avoided inaccuracies of spectral calibration associated with the use of many different instruments across the studies incorporated into the meta-analysis. In the histogram each subset is represented by a rectangle whose height is equal to the count of observations that fall into the wavelength interval. A quantile plot is a simple and effective way to compare different wavelength distributions. Let *λ*_*i*_ (*i* = 1to*G*) be the wavelengths sorted in increasing order so that *λ*_1_ is the smallest wavelength and *λ*_*G*_ is the largest. Each wavelength, *λ*_*i*_, is paired with a percentage, *f*_*i*_, which indicates that approximately 100 *f*_*i*_% of the data are below or equal to the value, *λ*_*i*_.

$$f_{i}=\frac{i-0.5}{G}(i=1,\ldots ,G)$$ (8)

In a quantile plot, *λ*_*i*_ is graphed against *f*_*i*_. This allows us to compare different wavelength distributions based on their quantiles [114].

## Results

### 3.1 Quantifying the development of remote sensing of plant pigment concentrations

**Fig 2:**
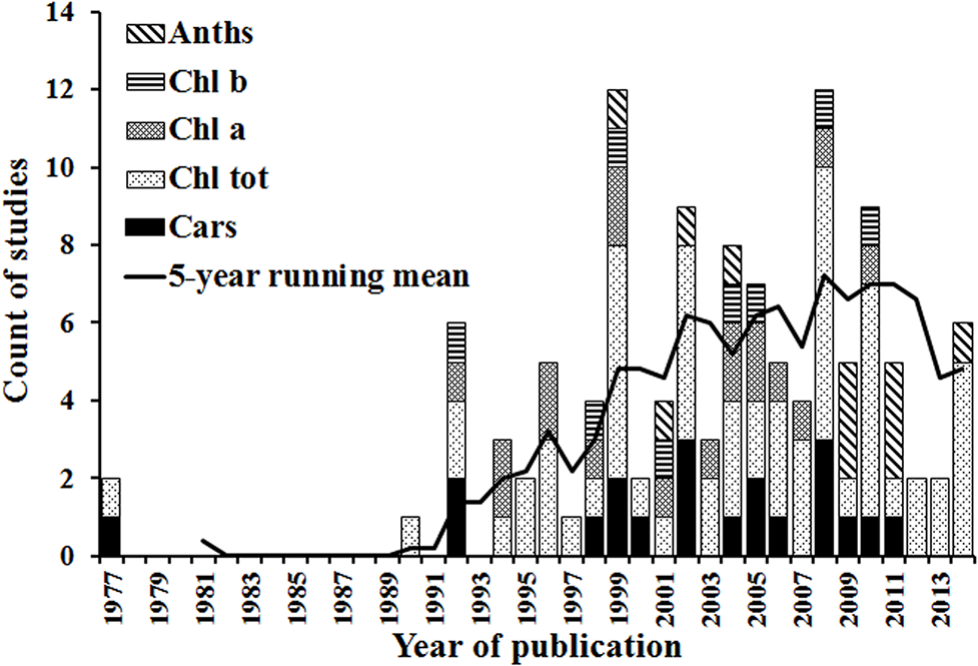
Histogram of numbers of selected studies published over time, showing the total in each year and the number focusing on each pigment type. The solid line is a 5-year running mean of the total number of studies.

The number of studies used in the meta-analysis published over the period from 1977 to 2014 are shown in Fig 2, along with the 5-year running mean which summarises the overall trajectory of development in this scientific field. After the first two studies were published in 1977 there were no other publications for 11 years, but then there was fast rate of growth from 1990 to 1999. The number of publications reached top in 1999 after which the publication rate stopped increasing, indicating that research in passive optical hyperspectral remote sensing of plant pigment concentrations is within a mature phase. The overall trajectory of publications shows three periods covering the origins, development and proliferation of research in this field. This trajectory corresponds to the developmental phases of hyperspectral instruments, which started with spectrophotometers and hand-held spectroradiometers enabling leaf and canopy-scale work. With the more recent advent of airborne and spaceborne imaging spectrometers, more landscape scale analyses have become possible.

Despite this overall development in the field, there were substantial differences in research on different pigments. The first studies of total chlorophyll and carotenoids were published in 1977, followed by chlorophyll *a* and chlorophyll *b* in 1992 and anthocyanins in 1999. The growth rate of publications on chlorophyll *a*, chlorophyll *b*, carotenoids and anthocyanins has been significantly lower than that for total chlorophyll. These differential rates of growth are perhaps indicative of the increased difficulty in quantifying the concentrations of individual photosynthetic and protective pigments remotely.

### 3.2 The relationships between pigment concentrations and remotely sensed variables

**Fig 3:**
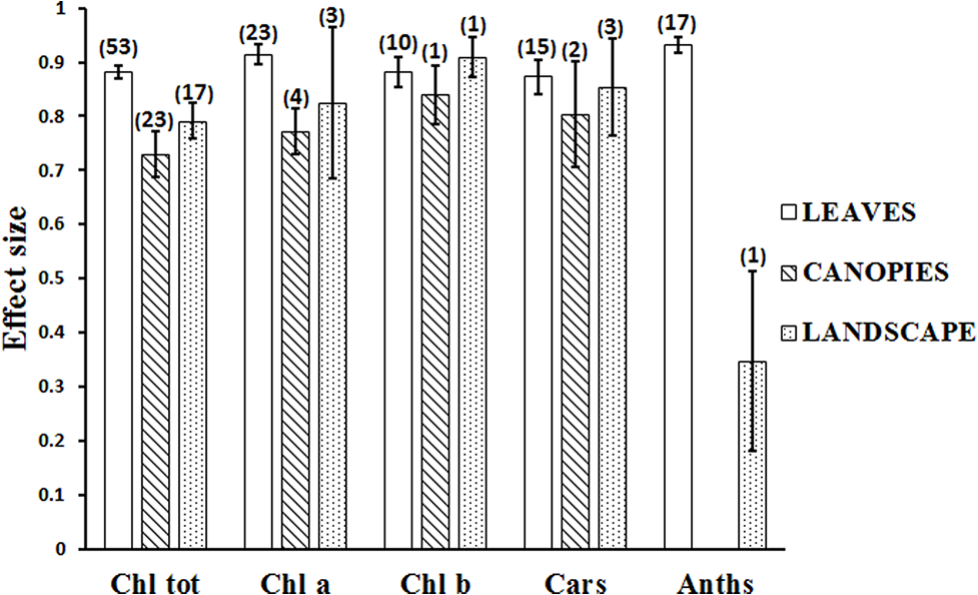
The mean effect size for pigment types at the scales of leaf, canopy and landscape. (The numbers of reported relationships found in the literature are shown in brackets, error bars represent 95% confidence intervals).

The mean effect size for different pigments at the scales of leaf, canopy and landscape were calculated (Fig 3). At the leaf scale, the mean effect sizes were fairly consistent between different pigment types, varying from 0.87 to 0.93, while the difference in mean effect sizes between pigment types was statistically significant at the canopy and landscape scales. The mean effect size presented the highest value 0.93 (95% confidence interval, 0.92–0.95) for anthocyanins quantification at the leaf scale, far higher than the result of 0.35 (95% confidence interval, 0.18–0.51) at the landscape scale. The mean effect size for total chlorophyll quantification was 0.88 (95% confidence interval, 0.87–0.89) at the leaf scale, 0.73 (95% confidence interval, 0.69–0.77) at the canopy scale and 0.79 (95% confidence interval, 0.76–0.82) at the landscape scale. The mean effect size for carotenoids was the lowest of the various pigments at 0.87 (95% confidence interval, 0.84–0.90) at the leaf scale, still higher than the result 0.80 (95% confidence interval, 0.71–0.90) at the canopy scale and 0.85 (95% confidence interval, 0.76–0.94) at the landscape scale. The results show that these mean effect sizes varied across pigment types and scales. In general, the relationships are stronger at the leaf scale than those at the canopy and landscape scales.

Fig 3 shows that the highest number of relationships published was for pigment quantifications at the leaf scale. Pigment quantification at the canopy scale was less frequently reported in the literature and only a few studies were conducted at the landscape scale. This can be attributed to the limited availability and high costs of suitable airborne and spaceborne hyperspectral instruments [20]. For each scale, the highest number of relationships published was for total chlorophyll quantification, followed by chlorophyll *a*, carotenoids, chlorophyll *b* and anthocyanins. These findings are consistent with previous studies [10,11].

### 3.3 Wavelength selection for pigment quantification using remotely sensed data

#### 3.3.1 Optimal wavelengths for chlorophyll quantification

**Fig 4:**
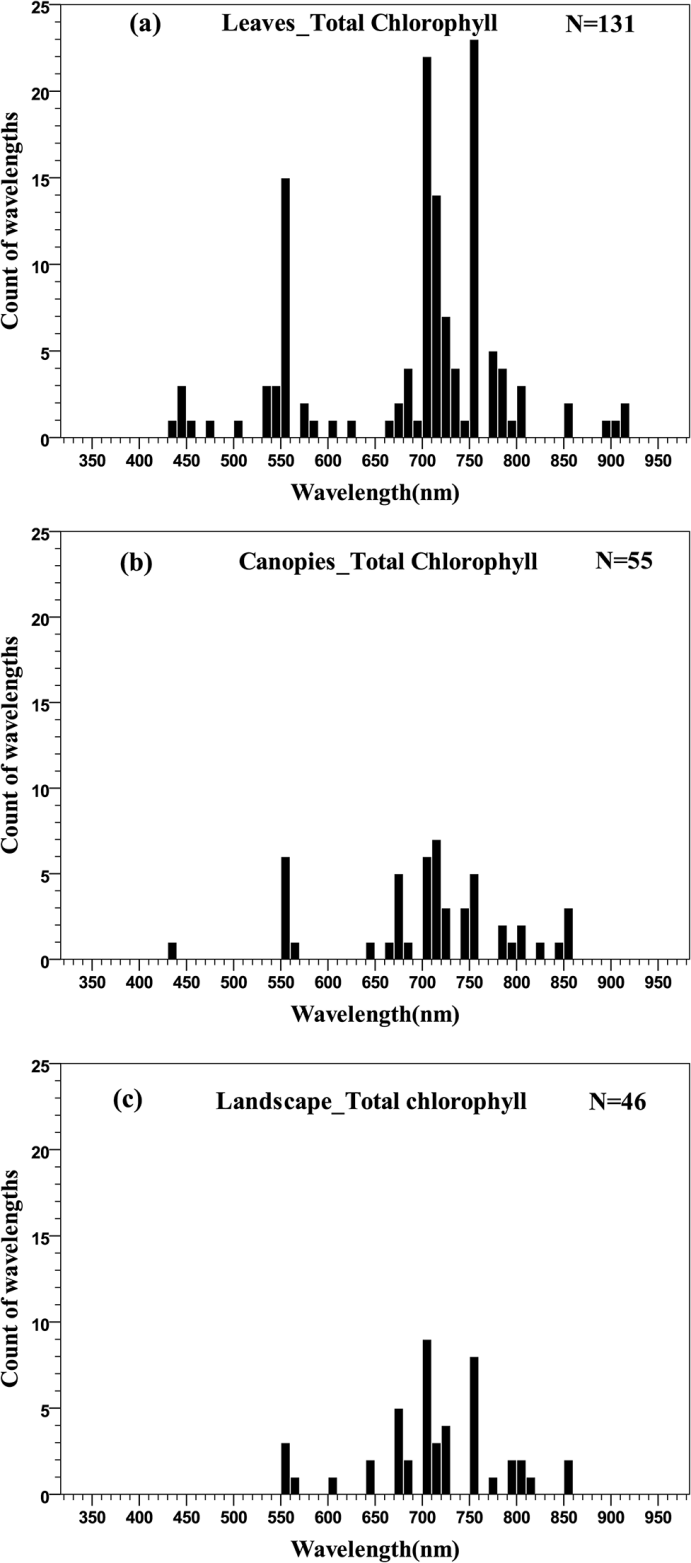
Histogram of wavelengths for total chlorophyll quantification using remotely sensed data at leaf (a), canopy (b) and landscape (c) scales using an interval width of 10 nm.

There is a large quantity of studies on the relationships between chlorophyll concentration and remotely sensed data. The distributions of wavelengths used at the three scales are shown in Fig 4. It should be noted that all of the wavelengths for pigment quantification were concentrated in the 350–950 nm region, except for total chlorophyll quantification at the canopy scale, which spread over 400–2400 nm. For comparison, wavelengths in the histograms and quantile plots were limited within the 350–950 nm region.

**Fig 5:**
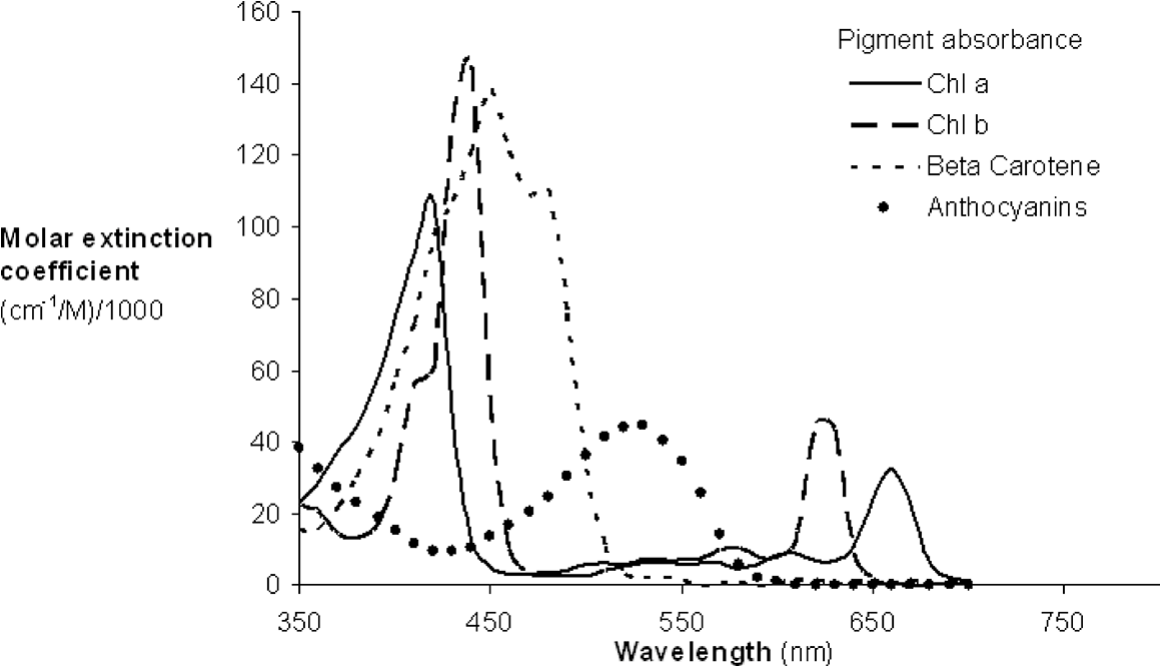
Absorption spectra of the major plant pigments (reproduced from Blackburn, 2007).

In general, the distribution of wavelengths displayed a double-peak feature, concentrated in the green (550–560 nm) and red edge (680–750 nm) regions rather than the main absorption wavelengths of chlorophyll (blue or red) (Fig 5). At the canopy scale, five wavelengths in the NIR to SWIR regions (1000–2400 nm) were also used for total chlorophyll quantification (not shown in Fig 4B). This is due to the major influence of canopy structure in canopy reflectance and because leaf chlorophyll concentration was relatively stable in the particular studies [76,77].

**Fig 6:**
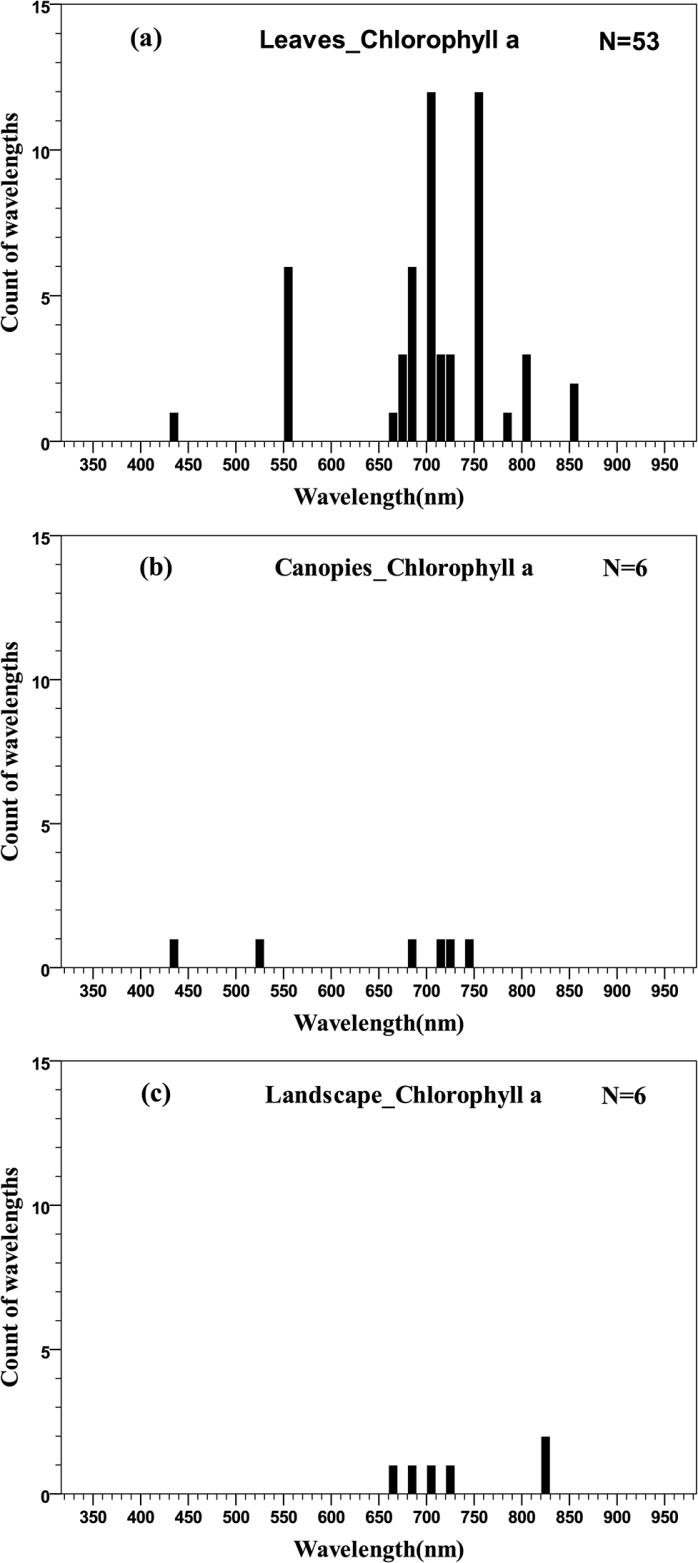
Histogram of wavelengths for chlorophyll *a* quantification using remotely sensed data at leaf (a), canopy (b) and landscape (c) scales by an interval width of 10 nm.

The distribution of wavelengths proposed for chlorophyll *a* quantification at the leaf scale was similar to that of total chlorophyll, concentrated in the green and red edge ranges (Fig 6A). At the canopy and landscape scales, the number of wavelengths is limited and is difficult to identify the central tendency of wavelength distribution (Fig 6B and Fig 6C).

**Fig 7:**
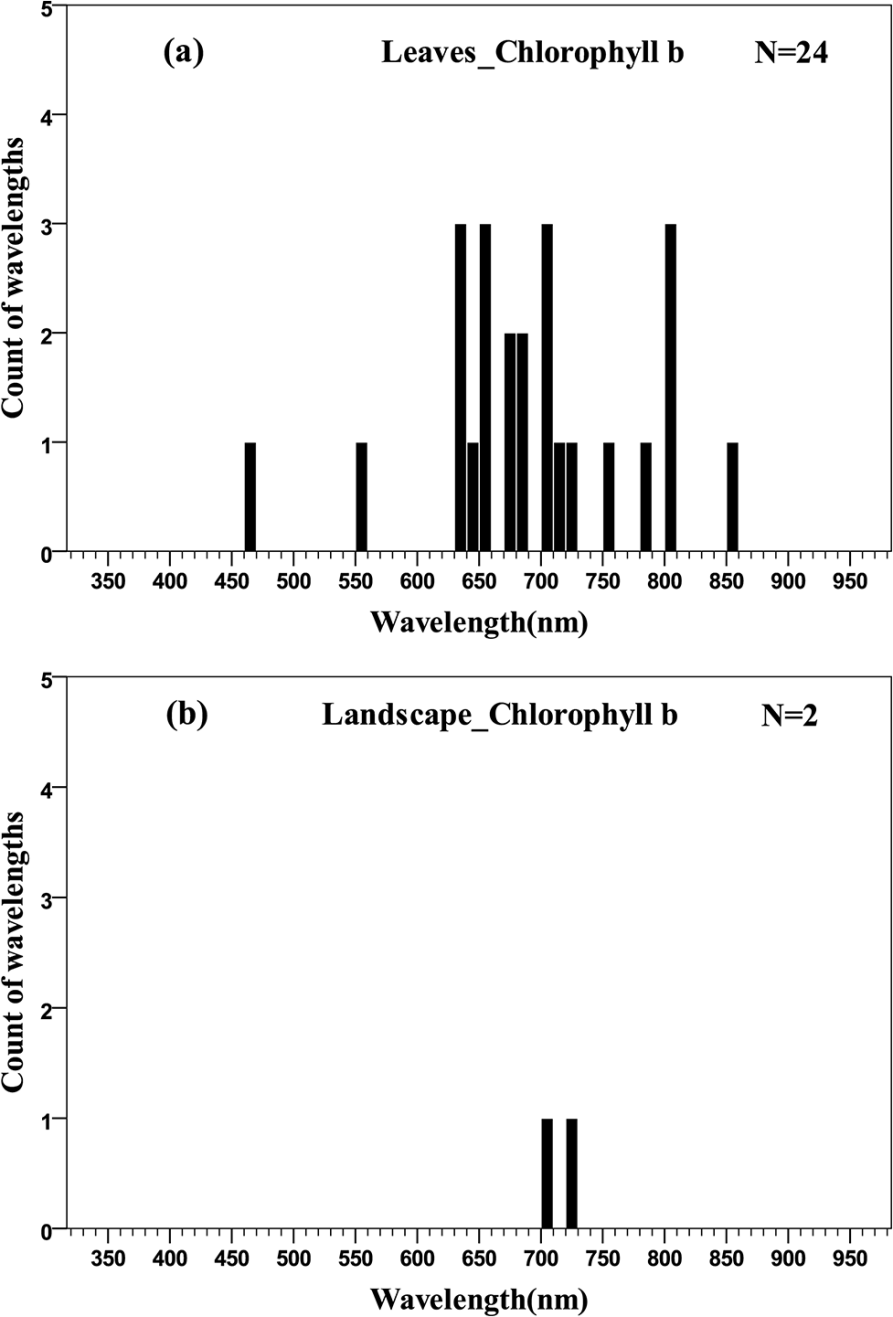
Histogram of wavelengths for chlorophyll *b* quantification using remotely sensed data at leaf (a) and landscape (b) scales using an interval width of 10 nm.

**Fig 8:**
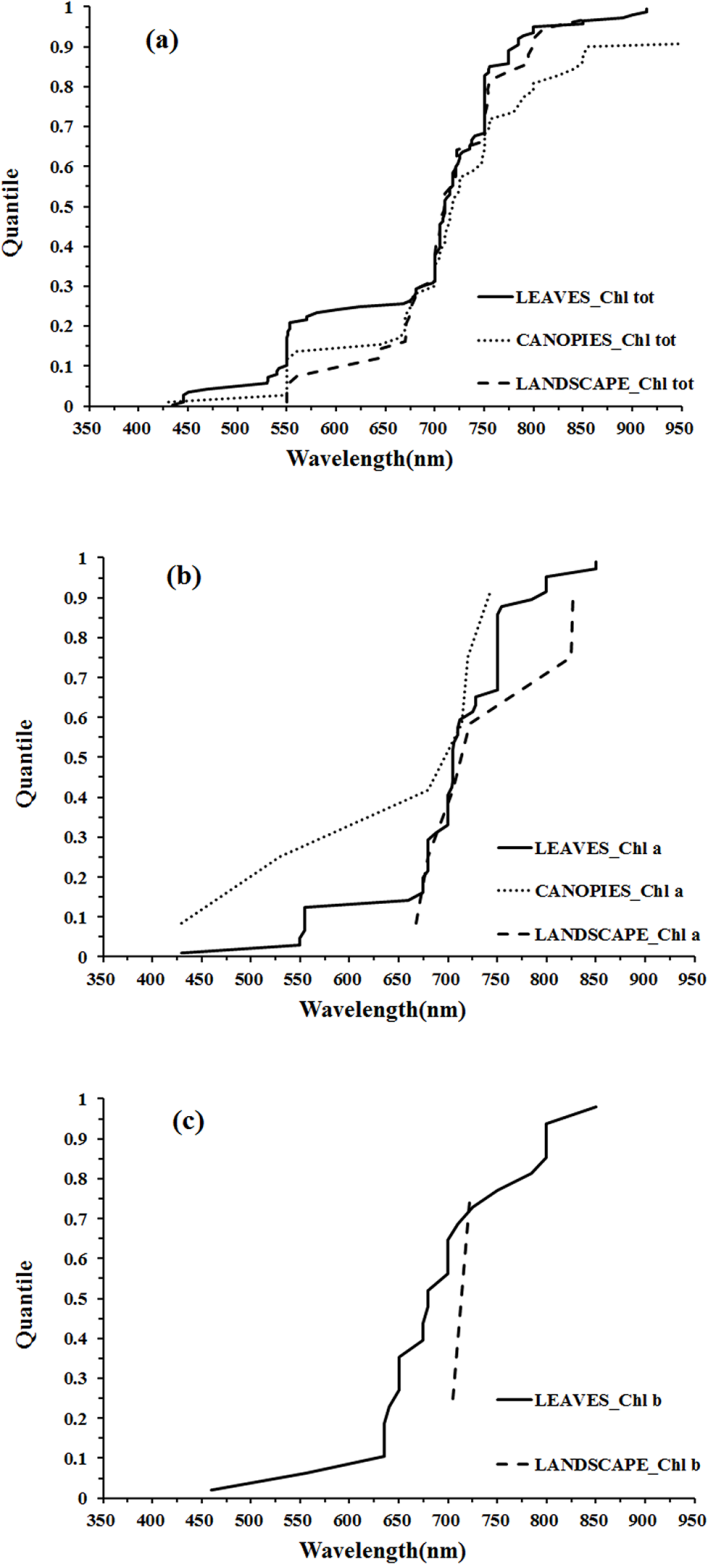
Quantile plots of the wavelengths used for the quantification of Chl tot (a), Chl a (b), and Chl b (c) at different scales.

The distribution of wavelengths used for chlorophyll *b* quantification at the leaf scale were concentrated in the main absorption wavelength of chlorophyll *b* (red, 630–660 nm), the red edge (670–710 nm) and the NIR (800–810 nm) regions (Fig 7A). Only two wavelengths were selected at the landscape scale and could not be used for statistical inference (Fig 7B). The distributions of wavelengths used for quantification of different pigments at different scales can be compared in the quantile plots (Fig 8). There were similar wavelength distributions for total chlorophyll quantification at the scales of leaf, canopy and landscape (Fig 8A). For chlorophyll *a* there were similar wavelength distributions at the leaf and canopy scales, but the landscape scale differed (Fig 8B), while a comparison across scales for chlorophyll *b* was difficult due to a lack of data at scales other than the leaf (Fig 8C).

**Fig 9:**
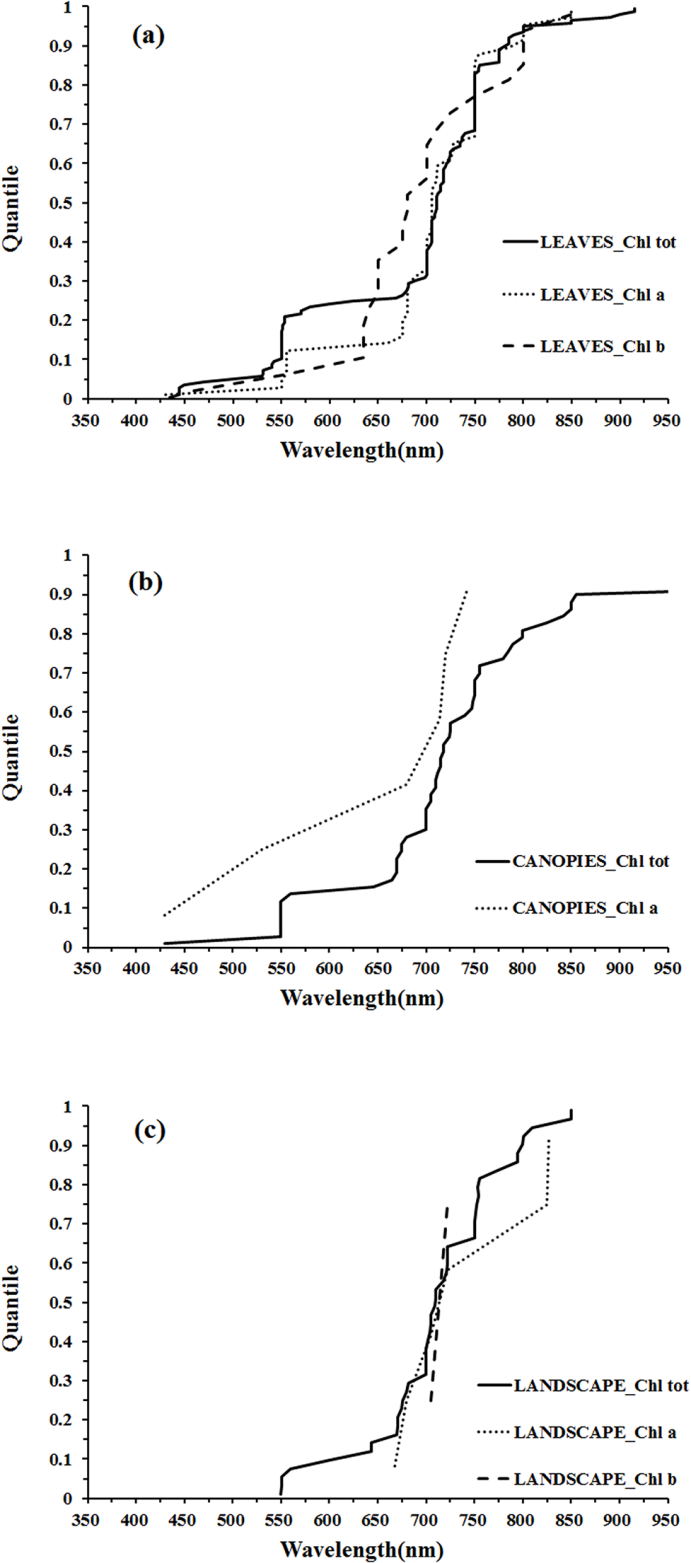
Quantile plot of the wavelengths used at leaf (a), canopy (b) and landscape (c) scales for the quantification of Chl tot, Chl a, and Chl b.

At the leaf scale, the wavelength distributions for total chlorophyll and chlorophyll *a* quantification were relatively similar while there were notable differences for chlorophyll *b* (Fig 9A). In the region 425–625 nm, the wavelengths used for chlorophyll *a* quantification were concentrated in the region of the green peak in leaf reflectance (550nm), but the central tendency of wavelength distribution for chlorophyll *b* quantification was not obvious. In the red region, the wavelength distribution for chlorophyll *a* quantification was shifted to longer wavelengths than that of chlorophyll *b* (Fig 9A). The significant overlap in the absorption features of chlorophyll *a* and chlorophyll *b* (Fig 5) and the low concentrations of chlorophyll *b* with respect to chlorophyll *a* in most leaves can present difficulties in defining optimal wavelengths for chlorophyll *b* quantification. The absorption spectra of chlorophyll *a* and chlorophyll *b* both display a double-peak feature; the absorption maxima of chlorophyll *a* are at 430 and 662 nm, and chlorophyll *b* has peaks located at 453 and 642 nm (Fig 5). In the presence of carotenoids, it is difficult to separately assess chlorophyll *a* and chlorophyll *b* from reflectance data in the blue region. However, in the red region, the wavelength position of maximum absorption by chlorophyll *a* is longer than that of chlorophyll *b*, which can be exploited for chlorophyll *a* and chlorophyll *b* discrimination (as seen in Fig 9A). The capacity to use this approach to discriminate chlorophyll *a* and chlorophyll *b* is difficult to assess at the canopy and landscape scales due to the small number of studies on chlrophyll b (Fig 9B and 9C).

#### 3.3.2 Optimal wavelengths for carotenoids quantification

**Fig 10:**
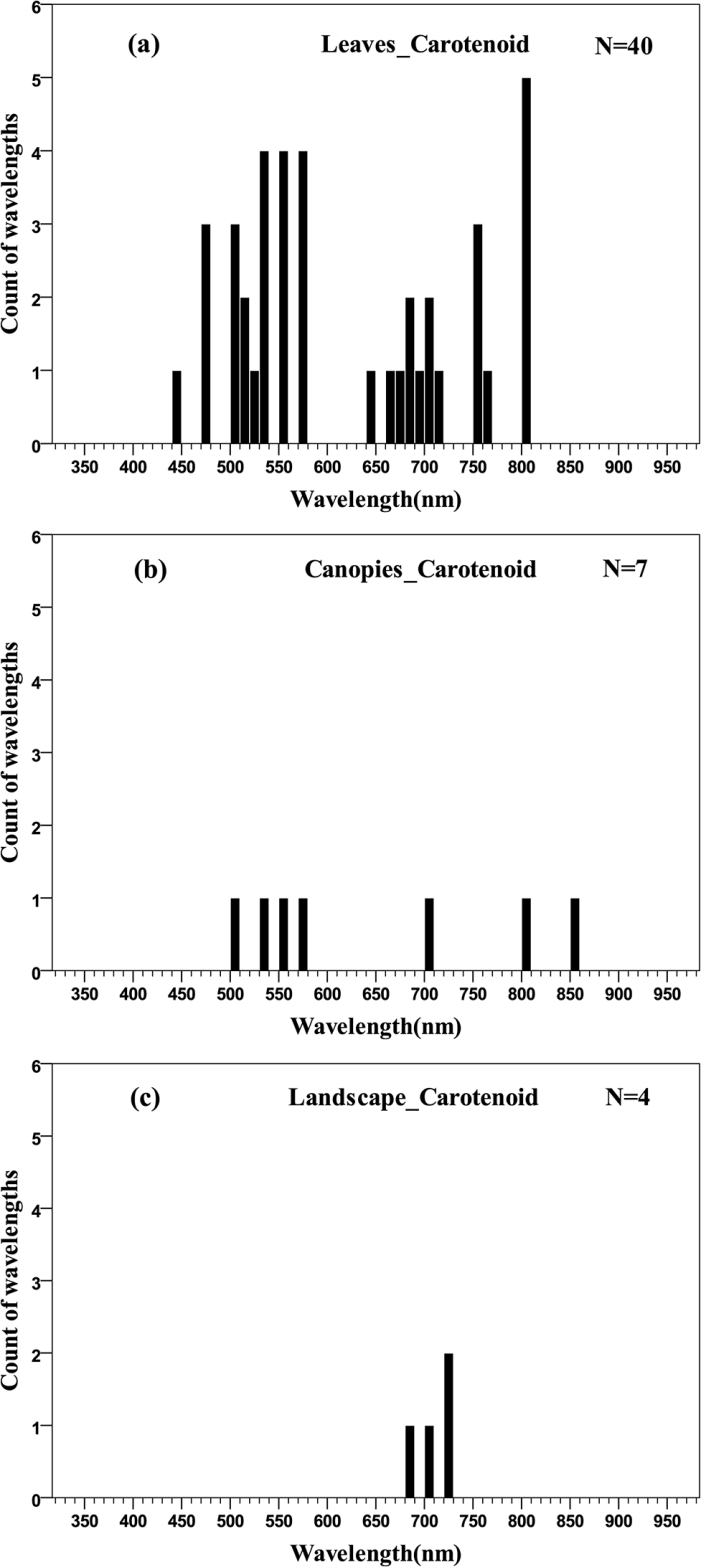
Histogram of wavelengths for carotenoids quantification using remotely sensed data at leaf (a), canopy (b) and landscape (c) scales using an interval width to 10 nm.

**Fig 11:**
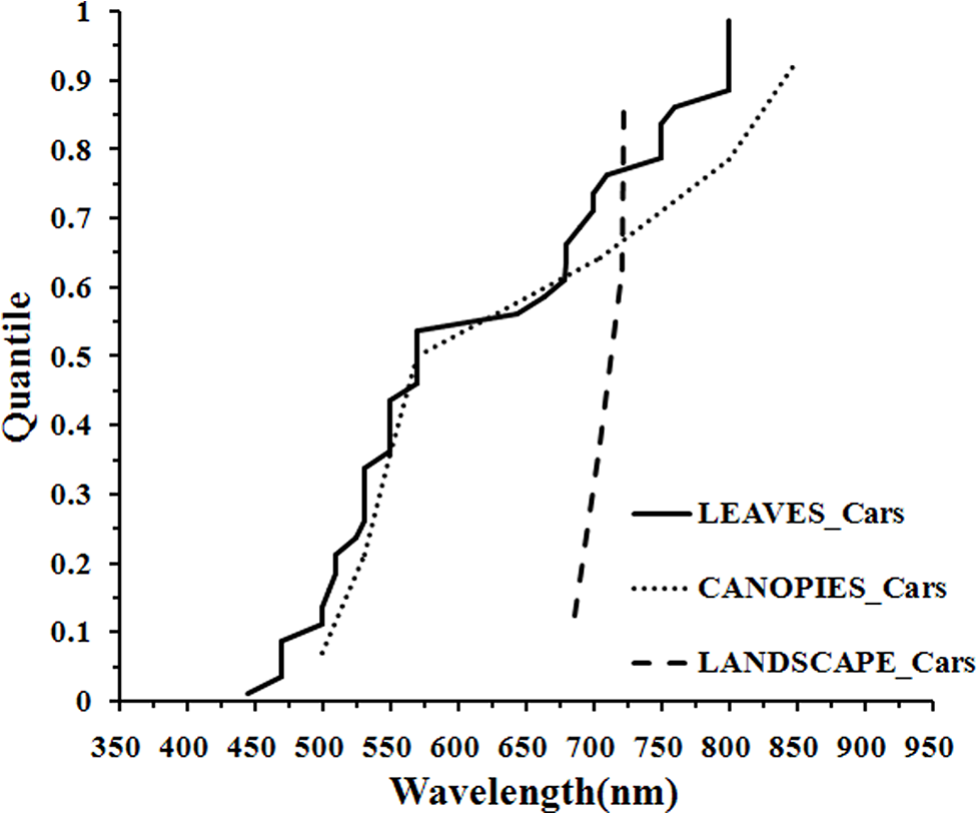
Quantile plot of the optimal wavelength for the quantification of Cars at different scales.

At the leaf scale, the central tendency of wavelength distribution was not obvious but was mainly concentrated in the 500–580 nm region (Fig 10A). There were similar wavelength distributions for carotenoids quantification at the leaf and canopy scales (Fig 11) but at the landscape scale, the number of wavelengths was too small for statistical inference (Fig 10C).

#### 3.3.3 Optimal wavelengths for anthocyanins quantification

**Fig 12:**
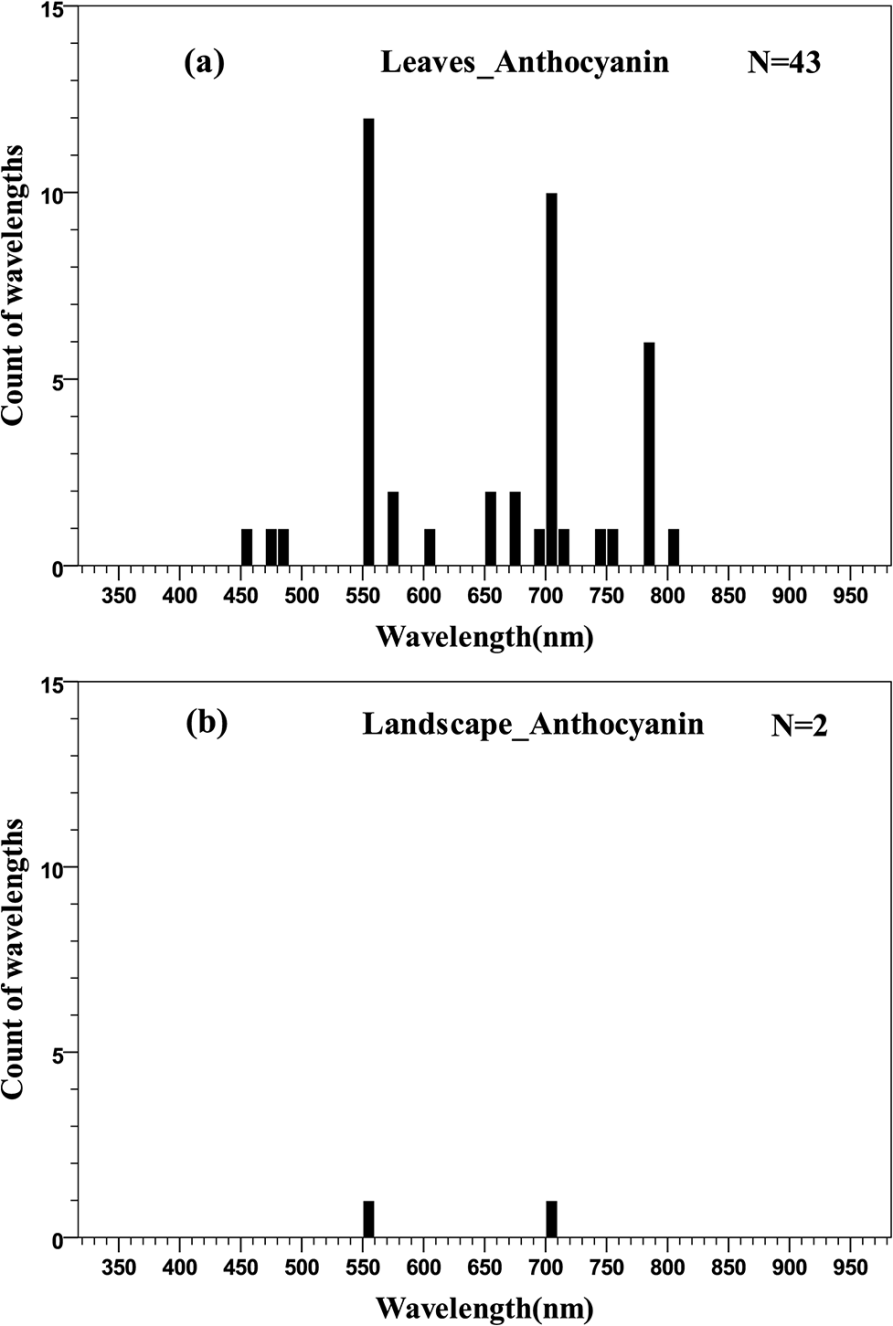
Histogram of wavelengths for anthocyanins quantification using remotely sensed data at leaf (a) and landscape (b) scales using an interval width to 10 nm.

**Fig 13:**
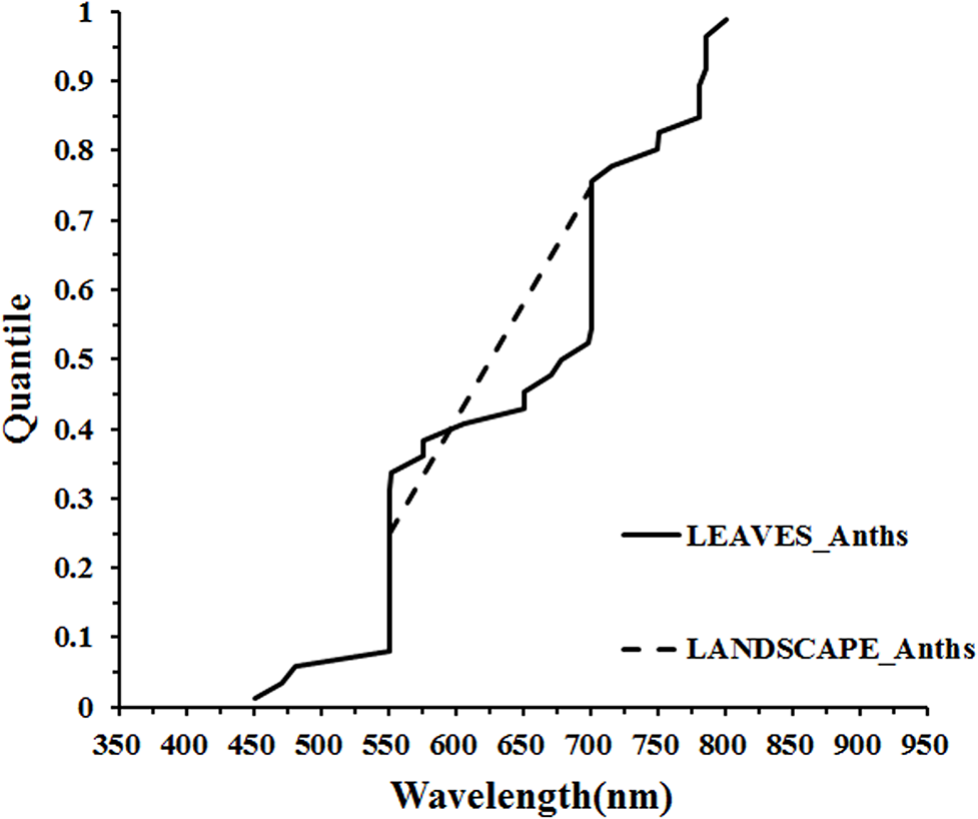
Quantile plot of the optimal wavelengths for the quantification of Anths at different scales.

Quantification of anthocyanins from reflectance data has been given less attention by the passive optical hyperspectral remote sensing community than chlorophyll and carotenoids. Most studies have concentrated on the quantification of anthocyanins at the leaf scale, with some work at the landscape scale but nothing at canopy level. At the leaf scale, the distribution of wavelengths used for quantifying anthocyanins was concentrated in the main absorption wavelength of anthocyanins (green, 550–560 nm), the red edge (700–710 nm) and the NIR (780–790 nm) ranges (Fig 12A). Similarly, the two wavelengths used to estimate anthocyanin concentration at the landscape scale were distributed in the green and red edge regions, respectively (Fig 12B and Fig 13).

## Discussion

This meta-analysis of 85 studies has demonstrated that remotely sensed variables are good estimators of plant pigment concentration. Most of the studies were conducted at the leaf scale, while pigment quantification at the canopy and landscape scales was less frequently reported. For each scale, most of the studies were conducted for total chlorophyll quantification, followed by chlorophyll *a*, carotenoids, chlorophyll *b* and anthocyanins. These findings are consistent with previous studies [10,11].

The strength of these relationships varied across pigments types and scales. In general, the relationships are stronger at the leaf scale than those at the canopy and landscape scales. At the leaf scale, the mean effect sizes were fairly consistent across different pigment types and were all greater than 0.87, while the difference in mean effect sizes between pigment types was statistically significant at the canopy and landscape scales. This result has been widely assumed, yet a quantitative evaluation has been lacking. At the leaf scale, the methodological basis for pigment quantification has been fully explored, which provides an important basis for developing estimation models at the canopy and landscape scales. The primary goal of most leaf scale passive optical hyperspectral remote sensing studies has been to develop analytical approaches for pigment quantification that can be applied to data from airborne and spaceborne sensors [11].

At the canopy and landscape scales, the experimental results are influenced by a number of factors, which obscures the relationships between spectral reflectance and concentrations of individual pigments. The reflectance spectrum of a whole canopy is subject to canopy biophysical attributes (e.g., orientation of leaves (leaf angle distribution; *LAD*), variations in number of leaf layers (*LAI*) and foliage clumping), presence of non-leaf elements (e.g., soil reflectance and the proportions of shadowed and sunlit background), anisotropic scattering of photons to interact with multiple surfaces such as leaves, woody material and soils, viewing geometry (e.g., sun and view zenith and azimuth angles) and illumination conditions (e.g., the ratio between direct and diffuse sunlight and atmospheric condition). It is the interaction of these factors, including their potential covariance or unique behavior that drive variation in canopy and landscape reflectance characteristics in three-dimensional space [10,22].

It should be noted that part of the variability in effect sizes at the canopy scale may be entirely artifactual. These artifacts are common in experimental studies: studies vary in terms of the quality of measurement; researchers make computational errors; people make typographical errors in copying numbers from handwritten tables to computer; and sampling errors. With the advent of airborne and spaceborne imaging spectrometers, there have been opportunities to measure plant pigment concentrations at the landscape scale. The reflectance spectrum from airborne and spaceborne sensors is subject to even more controlling factors, notably, soil/litter surface reflectance, and vegetation structure. The range of controlling factors should be taken into account in subsequent analyses.

Table 2 shows that the total sample size at the leaf scale is much more than that of canopy and landscape scales. The law of large numbers correctly states that large samples are reasonable representations of the population and parameter estimation is close to the real values when the sample size is large enough. Many researchers seem to believe that the same law applies to small samples and severely underestimate the amount of variability in findings that is caused by sampling errors. As a result, they erroneously expect statistics based on small samples to be close to the real values [13]. At the canopy and landscape scales, the number of studies and total sample size is limited, which influences the robustness and accuracy of effect sizes.

Despite the significant difference in effect sizes between different scales, it was found that the wavelength distribution for total chlorophyll quantification at the scales of leaf, canopy and landscape was similar, being concentrated in the green (550–560 nm) and red edge (680–750 nm) regions rather than the main absorption wavelength of chlorophyll (blue or red). The consistency in optimal wavelengths across scales can be attributed to several factors: (1) despite the many factors influencing reflectance at the canopy and landscape scales, it is the selective absorbance properties of pigments that determines the selection of wavelengths for pigment quantification, and (2) several estimation models derived at the leaf scale were directly applied to canopy and landscape scales. This suggests that the leaf-level study has provided an important basis for developing estimation models at the canopy and landscape scales.

At the leaf scale, the distribution of wavelengths used for chlorophyll *a* quantification was similar to that of total chlorophyll; the distribution of wavelengths for chlorophyll *b* quantification was concentrated in the main absorption wavelength of chlorophyll *b* (red, 630–660 nm), the red edge (670–710 nm) and the NIR (800–810 nm) regions; the central tendency of wavelength distribution for carotenoids quantification was not obvious, but was mainly concentrated in the 500–580 nm region; for the estimation of anthocyanins, the distribution of wavelengths was concentrated in the main absorption wavelength of anthocyanins (green, 550–560 nm), the red edge (700–710 nm) and the NIR (780–790 nm) ranges. In the present meta-analysis, the lack of studies reporting the quantification of carotenoids and anthocyanins at the canopy and landscape scales has hindered cross-scale comparisons (Fig 10; Fig 12). Consequently, it is not entirely clear if the optimal wavelengths for carotenoids and anthocyanins quantification at the leaf scale are necessarily the optimal wavelengths at the canopy and landscape scales, where multiple scattering and other confounding effects may alter the spectral response of individual pigments, much in the way that pigment absorption peaks can vary depending upon their chemical and scattering medium. Therefore, more work may be needed to determine the optimal algorithms for airborne or spaceborne platforms.

It should be noted that the lack of statistical information in the studies (e.g., sample size and coefficient of determination) has hindered a more comprehensive cross-study comparison in the present research. When selecting the final set of studies, 50 studies were excluded due to the lack of statistical information. Insufficient statistical information can not only limit the research population covered by meta-analysis but also render the findings of the original study somewhat suspect. Thus, it is suggested that when conducting primary research, such information should include, but not be limited to, the sample size, the pertinent test statistic (e.g., r, t, or F), the unit of pigment concentration/content, the range of pigment concentrations/content, and estimation precision for pigment quantification (e.g. root mean squared error, RMSE).

This study has established the possibility of integrating the results of studies on the passive optical hyperspectral remote sensing of plant pigment concentrations across a range of vegetation types and scales using a meta-analysis approach. Despite the robust models for pigment prediction at the leaf scale, the continuing challenge is to properly account for the multiple factors introduced by scene components such as sunlit and shaded parts of tree crowns and gaps influencing the retrieved signal at the canopy and landscape scales. Recent work have illustrated that, in addition to other influencing factors such as illumination geometry and atmospheric conditions, canopy architecture had an important control on the applicability of models for pigment prediction. Scanning LIDAR systems have only recently become widely available which enable the estimation of the range between the sensor and a target by recording the time during which the emitted laser pulse is reflected off an object and returns to the sensor [21]. LIDAR systems have the ability to directly measure spatial variations in canopy height and other aspects of the vertical structure of canopies. Given the high degree of structural complexity at the canopy and landscape scales, it would appear that the integration of vertical canopy structural information provided by active LIDAR remote sensing with hyperspectral reflectance may has both a structural and physiological interpretation and improve the estimation of pigment concentrations over passive optical hyperspectral imagery alone [102].

## Supporting Information

S1 PRISMA Checklist PRISMA (Preferred Reporting Items for Systematic Reviews and Meta-Analyses) Checklist. (DOC)
